# Hyperoxia during venoarterial ECMO: Culprit or co-variate? A comment from the BLENDER investigators

**DOI:** 10.1186/s13054-022-04213-8

**Published:** 2022-11-08

**Authors:** Lavienraj Premraj, Alastair Brown, Aidan Burrell, John F. Fraser, David Pilcher

**Affiliations:** 1 Griffith University School of Medicine, Gold Coast, QLD Australia; 2 Critical Care Research Group, The Prince Charles Hospital, Chermside, QLD Australia; 3 Department of Epidemiology and Preventive Medicine, Australian and New Zealand Intensive Care Research Centre, School of Public Health, Monash University, Melbourne, Australia; 4 Department of Intensive Care, Alfred Health, The Alfred Hospital, Commercial Road, Melbourne, VIC 3004 Australia; 5 Faculty of Medicine, University of Queensland, Brisbane, Australia; 6 Australian Centre for Health Services Innovation (AusHSI) and Centre for Healthcare Transformation, School of Public Health and Social Work, Queensland University of Technology (QUT), Brisbane, Australia; 7 St Andrew’s War Memorial Hospital, UnitingCare, Spring Hill, Australia; 8 The Australian and New Zealand Intensive Care Society (ANZICS), Centre for Outcome and Resources Evaluation, Melbourne, Australia

The clinical significance of hyperoxia in critically ill patients remains unclear [1–3]. Oxidative stress has been linked to the progression of numerous disease states. It can be defined as an imbalance between the production of reactive oxygen species (ROS) and anti-oxidant capacity resulting in damage of cellular components. Exposure to supraphysiological partial pressures of oxygen can induce oxidative stress [4]. Interpretation of observational studies and clinical trials is exceedingly challenging due to heterogeneity brought about by patient and disease factors [3, 5].

Patients requiring Venoarterial extracorporeal membrane oxygenation (VA-ECMO), may be at increased risk from hyperoxia due to pre-existing oxidative stress from ischemia reperfusion injury and a high prevalence of extreme hyperoxia [6, 7]. As such the control and effect of hyperoxia exposure during VA-ECMO requires investigation. The Extracorporeal Life Support Organization (ELSO) guidelines caution against excessive hypo- and hyper-oxemia, recommending “slight hyperoxemia after the oxygenator (150 mmHg)” [8]. However, achieving this is complicated by the many parameters influencing oxygenation during VA-ECMO. Arterial partial pressure of oxygen (PaO_2_) results from the interplay between multiple factors: fraction of oxygen delivered by the ventilator (FiO_2_), fraction of oxygen in sweep gas delivered to the membrane (F_b_O_2_), ventilation: perfusion (V/Q) matching in the lung and the balance of ECMO flow and native cardiac output.

Several observational studies have examined the association between hyperoxia during VA-ECMO and patient outcomes and the findings are inconsistent [6, 9–12]. The methodology of these studies have been highly variable and although the demonstrated effect of hyperoxia has varied a high prevalence of hyperoxia amongst VA-ECMO patients has been ubiquitous [7, 9–12].

In response to this question we consider the following:

1. *Interactions between ECMO and patient physiology* High PaO_2_, particularly measured at the right radial artery, may reflect poor native cardiac output. In these cases, the mixing point is proximal to the innominate artery with high PO2 levels reflecting hyperoxic ECMO blood flow in right radial blood samples. Few studies have meaningfully considered the interplay between VA-ECMO physiology and disease course [6, 9, 10]. Although studies have controlled for general measures of disease severity, this mechanism of confounding cannot be excluded. Notably, Bonneiman et al. examined whether the position of the arterial line affected the association between hyperoxia and outcome [10] and found it did not: PaO_2_ was higher in samples from the femoral artery but the presence of hyperoxia was still associated with mortality.
2. *Duration and timing of hyperoxia exposure* We appreciate that the use of aggregate (mean PaO_2_) or instantaneous (PaO_2_ peak or 24 h post-cannulation) measures is a pragmatic decision. Still, variability in the reporting of PaO_2_ is ultimately undesirable. The method used to measure hyperoxia have been reported inconsistently. Indeed, the largest study (775 VA-ECMO patients), which did not identify specific oxygen thresholds corresponding to harm, analysed a single post-cannulation PaO_2_ reading and 24 h, did not sufficiently account for disease severity or report location of the arterial line (due to limitations of source data) [9]. To elucidate meaningful relationships longitudinal measures of exposure are preferable.
3. *Timing of VA-ECMO initiation* Observational studies have demonstrated an association between shorter duration from shock or arrest to ECMO [13]. By contrast early hyperoxia has been shown to be independently associated with poor neurological outcome at discharge [6]. Despite its significance, adjusting for timing of hyperoxia itself, is technically challenging.
4. *Subgroup effects depending on indication* It is noteworthy that the risk of hyperoxia has been most apparent amongst recipients of ECMO cardiopulmonary resuscitation (ECPR) in the above studies [10, 11]. Indeed in a subgroup analysis of the ICU-ROX trial there was a signal to harm from hyperoxia amongst those with hypoxic ischaemic encephalopathy [14].

The recently published study by Moussa et al. addresses many of these concerns [7]. Unlike some previous studies, arterial oxygenation (PaO_2_) was primarily controlled using the oxygen-air blender and recorded for 48 h post-admission. Previous observational studies in this population lacked clear protocols for oxygen titration [6, 9–12]. This created issues with defining exposure and confounding by indication. In recognition of the later concern, Moussa et al. employ propensity weighting for the likelihood of developing hyperoxia. In doing so, they concluded that mean PaO_2_ was associated with 28-day mortality. Notably peak, and overall mean PaO_2_ had similar effect (adjusted odds ratio = 2.65 [95% CI = 1.79–6.07] vs. 2.85 [95% CI = 1.12–7.37] respectively). Questions remain around specific PaO_2_ thresholds at which hyperoxia becomes clinically significant and the importance of hyperoxia duration. These questions are of particular relevance as Moussa et al., like others, confirm a dose-dependent relationship between hyperoxia and morality [6, 7].

Differential exposure to hyperoxia, secondary to VA-ECMO configurations, has been rarely discussed. Subclavian cannulation, especially on the right side, predisposes cerebral hyperoxia. Meanwhile femoral (peripheral) cannulation, in those with concomitant pulmonary injury, risks cerebral and coronary hypoxia and, subsequently the development of differential hypoxia. Moussa et al. demonstrate no difference in 28-day mortality between subclavian and femoral configurations via sensitivity analysis. Long term cardiovascular and neurological outcomes remain to be investigated.

Ultimately randomised controlled trials are needed to establish the causal link between oxygen exposure and clinical outcomes in VA-ECMO patients. The BLENDER trial (NCT03841084) has recruited 214-patients to September 2022. It examines the number of ICU free days in patients exposed to a conservative oxygen strategy (combined manipulation of FbO_2_ and FiO_2_ with a target arterial oxygen saturation [SpO_2_] of 92–96%) compared the liberal group (in whom the FbO_2_ remained at 1.0 while the FiO_2_ was adjusted to SpO_2_ of 97–100%). Twice daily post oxygenator blood gases are being collected for seven days. Patients are randomised within 6 h of ECMO initiation, to limit hyperoxia exposure in the conservative arm and achieve meaningful group separation. Disability at 6 months is a key secondary outcome, particularly because hyperoxia has been associated with poor neurological outcome [6, 11]. Additionally, randomisation is stratified by ECPR status which will allows evaluation of the above parameters in this high-risk sub-group.

The management of oxygenation in critically ill patients remains a complex question. We commend the efforts of Moussa et al., and others that are helping untangle the narrative of hyperoxia in VA-ECMO; we eagerly await further evidence to guide clinical practice [15].

## Data Availability

NA.
